# Subcapsular Hepatic Hemorrhage: An Uncommon and Catastrophic Manifestation of Metastatic Melanoma

**DOI:** 10.7759/cureus.94001

**Published:** 2025-10-07

**Authors:** Akash Xavier, Sandra Toney, Hariharasudhan Balaji, Nithin Jyothy, Harsh Shah

**Affiliations:** 1 Emergency Medicine, Southern Health and Social Care Trust, Craigavon, GBR; 2 Radiology, St. John's Medical College Hospital, Bengaluru, IND; 3 Internal Medicine, Maidstone and Tunbridge Wells NHS Trust, Kent, GBR; 4 Radiology, Southern Health and Social Care Trust, Craigavon, GBR

**Keywords:** abdominal pain, acute presentation, diagnostic imaging, hepatic metastases, liver hemorrhage, malignant melanoma, multidisciplinary management, oncology emergency, palliative care, subcapsular hepatic hemorrhage

## Abstract

Subcapsular hepatic hematoma is a rare but potentially life-threatening manifestation of metastatic melanoma, often presenting with vague abdominal pain and risk of misdiagnosis. We report a 76-year-old man with a history of malignant melanoma who presented with acute epigastric pain radiating to the right shoulder, vomiting, and a syncopal episode. While initial blood tests showed mild leukocytosis and raised lactate, a CT aorta excluded dissection but revealed multiple hepatic metastases with a large subcapsular hematoma and pulmonary nodules. A follow-up CT mesenteric angiogram demonstrated interval hematoma expansion without active bleeding, further metastatic progression, and a segmental pulmonary embolus. Owing to frailty and advanced malignancy, a multidisciplinary team recommended conservative management. This case highlights the diagnostic conundrum of hepatic hemorrhage in melanoma, which may mimic other acute pathologies such as aortic dissection or acute coronary syndrome. Prompt cross-sectional imaging, serial monitoring, and early multidisciplinary input are essential for accurate diagnosis and for balancing intervention risk against quality of life in complex oncology presentations.

## Introduction

Metastatic melanoma is a malignancy characterized by its heterogeneous and often unpredictable patterns of dissemination [1]. Hepatic metastases occur in approximately 10-20% of patients with advanced-stage disease [2]. However, subcapsular hepatic hemorrhage resulting from metastatic spread is uncommonly rare in addition to being a potentially life-threatening complication [3,4]. Its nonspecific clinical presentation, which may include vague abdominal pain or signs of hemodynamic instability, poses a significant risk of misdiagnosis or delayed recognition [3,4]. Timely detection through imaging and urgent multidisciplinary intervention are critical to optimizing patient outcomes. Herein, we report a case of subcapsular hepatic hemorrhage secondary to rapidly progressive metastatic melanoma.

## Case presentation

A 76-year-old male with a history of malignant melanoma presented to the emergency department with a one-day history of acute, sharp epigastric pain radiating to the right shoulder, accompanied by two episodes of vomiting and a transient syncopal episode during triage. The presenting conditions of the patient necessitated immediate transfer to the resuscitation area. His medical history included malignant melanoma, iron deficiency anemia, chronic obstructive pulmonary disease, type 2 diabetes mellitus, chronic kidney disease, and essential hypertension. His regular medications comprised linagliptin, indapamide, perindopril, lercanidipine, and atorvastatin.

Initial clinical assessment revealed normal vitals, a Glasgow Coma Scale of 15/15 [5], and a National Early Warning Score of 0 [6]. Abdominal examination elicited mild epigastric tenderness on deep palpation. Laboratory investigations demonstrated mildly elevated white cell count, lactate, glucose, and troponin levels. Urea and electrolytes, CRP, and amylase levels were within normal limits. Electrocardiograms confirmed a normal sinus rhythm. He was prescribed ondansetron 4 mg orally and morphine 10 mg orally for symptom control.

**Figure 1:**
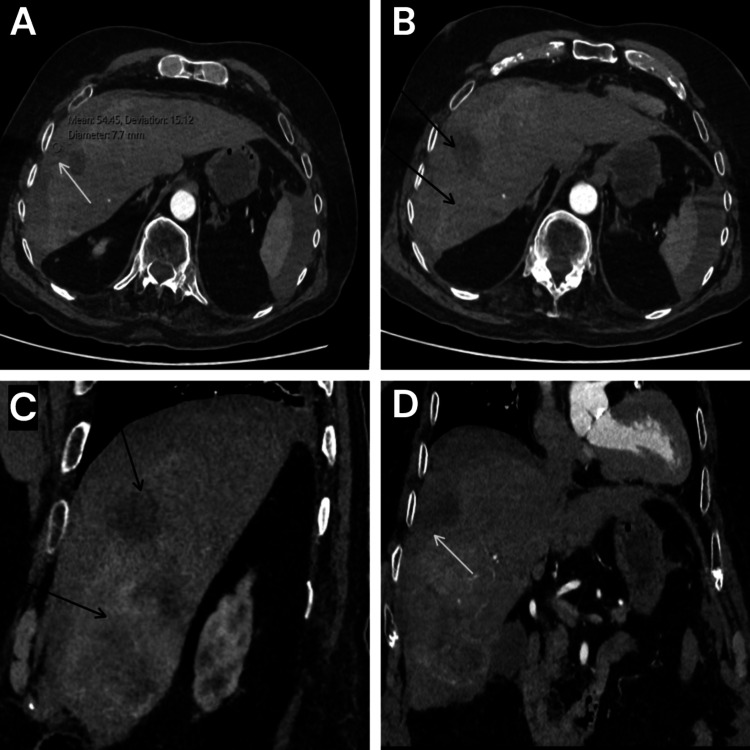
Contrast-enhanced CT of the aorta showing subcapsular hepatic hematoma and hepatic metastases (A) Axial CT view demonstrating high-density fluid in the subcapsular space of the right hepatic lobe, consistent with capsular breach and subcapsular hematoma (arrow). (B) Axial CT view showing multiple peripherally enhancing hepatic lesions consistent with metastases (arrows). (C) Sagittal CT view demonstrating multiple peripherally enhancing hepatic lesions consistent with metastases (arrows). (D) Coronal CT view demonstrating capsular breach with associated subcapsular hematoma (arrow).

**Figure 2:**
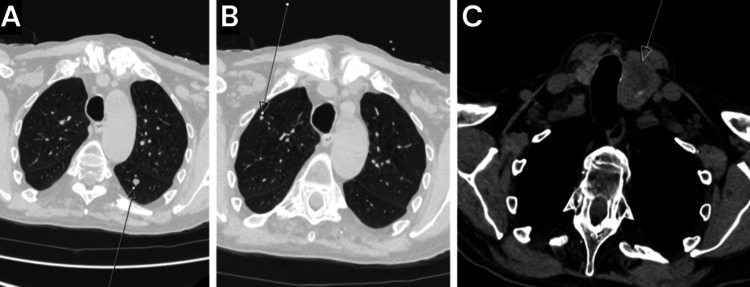
Contrast-enhanced CT of the aorta showing pulmonary and thyroid lesions consistent with metastatic disease (A) Axial CT view demonstrating a 6.1 mm pulmonary nodule in the left lower lobe, along with multiple other bilateral pulmonary nodules likely representing metastases (arrow). (B) Axial CT view showing a 5 mm pulmonary nodule in the right lung apex (arrow). (C) Axial CT view showing an enlarged left thyroid lobe with multiple nodules (arrow).

A contrast-enhanced CT scan of the aorta ruled out aortic dissection but identified multiple peripherally enhancing lesions in the liver consistent with hepatic metastases and high-density fluid in the subcapsular space of the right liver lobe, suggestive of capsular breach and subcapsular hematoma (Figure 1). Other findings included a bulky left lobe of the thyroid with multiple nodules, paraseptal and centrilobular emphysematous changes in the lungs, a 5 mm pulmonary nodule in the right lung apex, a 6.1 mm nodule in the superior segment of the left lower lobe, and multiple other bilateral lung nodules likely representing metastases (Figure 2). The patient was transferred to a tertiary center for monitoring for ongoing bleeding and evaluation for potential interventional radiology (IR) procedures.

**Figure 3:**
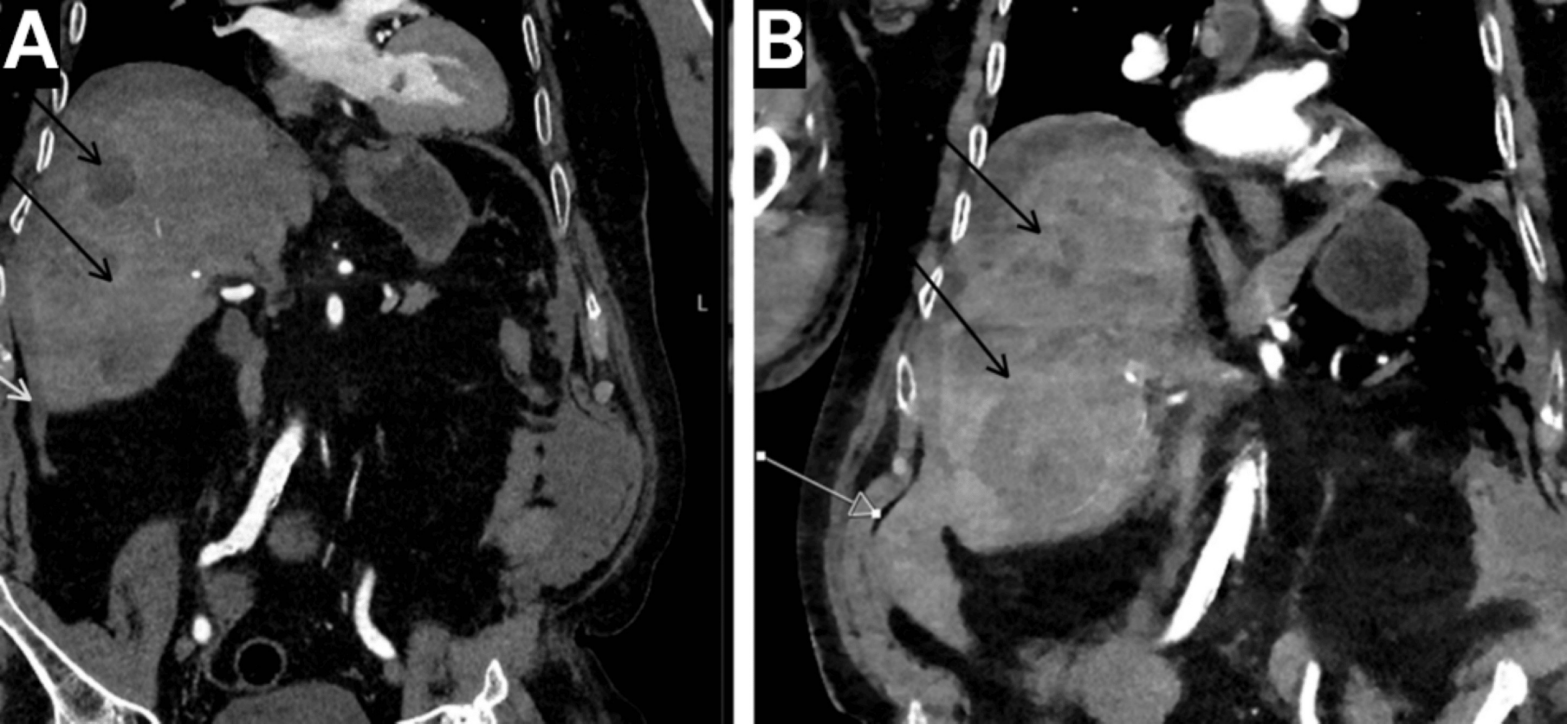
Interval progression on follow-up CT mesenteric angiogram compared with initial CT aorta (A) Contrast-enhanced CT aorta showing subcapsular hepatic hematoma (white arrow) and hepatic metastases (black arrows). (B) Follow-up CT mesenteric angiogram 10 days later, demonstrating expansion of the subcapsular hepatic hematoma (white arrow) and interval progression of hepatic metastases (black arrows).

During hospitalization, the patient developed an acute kidney injury with a declining estimated glomerular filtration rate, prompting the discontinuation of nephrotoxic medications (indapamide and perindopril). Despite elevated CRP and pyrexia, blood and urine cultures remained negative. An ultrasound of the urinary tract excluded hydronephrosis. A CT mesenteric angiogram revealed an increased volume of the subcapsular hepatic hematoma without evidence of active arterial bleeding or enlarging contrast blush, alongside a segmental pulmonary embolus in a lingular branch of the left pulmonary artery, a slight increase in small bilateral pleural effusions, and further progression of hepatic metastases, even compared to imaging from 10 days prior (Figure 3).

Given the rapid clinical deterioration and limited functional reserve, consultations with the acute oncology and palliative medicine teams led to a recommendation for best supportive care, including a trial of a short course of low-dose dexamethasone to manage fatigue and nausea. A do not attempt cardiopulmonary resuscitation (DNACPR) order was established, and symptom-directed medications were prescribed. The patient remained hemodynamically stable without requiring IR intervention and received a blood transfusion for a decline in hemoglobin. Interval imaging demonstrated significant progression of metastatic melanoma. He was subsequently transferred back to his local hospital for ongoing palliative care.

## Discussion

Hepatic metastases are common in advanced melanoma [2], yet spontaneous hemorrhagic presentations are exceptionally uncommon, with only isolated case reports documenting ruptured metastases leading to hemoperitoneum or subcapsular collections.

The liver’s extensive vascular network makes it prone to both metastatic spread and hemorrhagic complications [7]. This is particularly true for tumor invasion compromising the hepatic capsule or vasculature, leading to capsular breach and hematoma formation [7]. This risk is heightened in hypervascular tumors like melanoma metastases, which can erode vessels or cause intratumoral necrosis and bleeding [8].

The diagnosis in this case was established through a combination of clinical evaluation, laboratory investigations, and, most critically, cross-sectional imaging. The patient’s presentation with acute epigastric pain radiating to the right shoulder, suggestive of diaphragmatic irritation from subcapsular blood [9,10], along with vomiting and syncope, raised initial concerns in a patient with known melanoma. However, the nonspecific symptoms and stable hemodynamics initially delayed suspicion of a hemorrhagic event.

Laboratory findings, including mildly raised white cell count, lactate, and troponins, indicated systemic stress but were not diagnostic. Serial ECGs were normal, and inflammatory markers like CRP were initially unremarkable. The definitive diagnosis was made via contrast-enhanced CT of the aorta. A CT scan ruled out aortic dissection at the same time and also revealed a crescentic hyperdensity along the right liver lobe surface, indicative of subcapsular hematoma (Figure 1). In addition, there were new peripherally enhancing hepatic lesions (Figure 1) and multiple pulmonary nodules consistent with metastases (Figure 2). A follow-up CT mesenteric angiogram confirmed hematoma expansion without active extravasation (no contrast blush), further progression of metastases (Figure 3), and incidental findings like a segmental pulmonary embolus. These findings highlight the role of serial imaging in monitoring disease evolution in metastatic settings [8].

Differential diagnoses were systematically eliminated through targeted investigations. The epigastric pain and mild troponin elevation initially suggested acute coronary syndrome, but this was ruled out by normal sinus rhythm on serial ECGs and the absence of ischemic changes or chest pain progression. Peptic ulcer disease or gastritis was considered due to the pain’s location and vomiting, but normal amylase and CRP levels, along with no history of NSAID use or gastrointestinal symptoms, made this unlikely. Acute pancreatitis was excluded by normal amylase and the absence of typical risk factors or imaging evidence of pancreatic inflammation [11]. Aortic dissection was a key concern given the pain radiation and syncope but was definitively refuted by the CT aorta scan showing no dissection, intramural thrombus, or aneurysm. Other possibilities, such as biliary colic or perforated viscus, were dismissed due to the absence of jaundice, fever (initially), or free air on imaging. The rapid interval progression of metastases on imaging (within 10 days) further supported the malignant hemorrhagic complication rather than a static benign process (Figure 3).

Management of subcapsular hepatic hemorrhage varies based on clinical presentation and can range from conservative monitoring to IR procedures, such as embolization or surgical intervention [12]. In this case, conservative management was pursued due to the absence of active arterial bleeding (no contrast blush on imaging), the patient’s hemodynamic stability, and his overall palliative care trajectory amid advanced metastatic disease and comorbidities.

Complications during admission, including acute kidney injury (prompting discontinuation of nephrotoxic agents like indapamide and perindopril), pyrexia with negative cultures, and a hemoglobin drop requiring transfusion, were addressed supportively. The incidental pulmonary embolus was noted but likely not anticoagulated due to the bleeding risk. Multidisciplinary input was critical: acute oncology recommended best supportive care with low-dose dexamethasone for symptom control, palliative medicine instituted a DNACPR order and symptom-directed therapy, and physiotherapy addressed mobility decline from baseline independence to requiring assistance.

This holistic approach highlights the challenges in managing elderly patients with limited functional reserve, where aggressive interventions like transarterial embolization may be deferred to prioritize quality of life. Prognostically, such presentations in metastatic melanoma are associated with poor outcomes, with rapid disease progression and high mortality, emphasizing the importance of early oncologic surveillance in at-risk patients [13]. This case underscores the need for a coordinated approach involving vigilant monitoring of renal function, optimization of medication regimens, and input from surgical, oncology, and palliative care teams to navigate complex decision-making in resource-limited or end-stage scenarios.

## Conclusions

This case underscores the critical need to consider subcapsular hepatic hemorrhage as a rare but potentially catastrophic manifestation of metastatic melanoma. Atypical abdominal symptoms in patients with a history of malignancy, even in the absence of overt hemodynamic compromise, warrant prompt diagnostic imaging to facilitate early detection. Clinical vigilance, timely investigations, and robust multidisciplinary collaboration are paramount in ensuring appropriate management and delivering patient-centered care in such complex and challenging cases.
